# *Apc*-Mutant Kyoto Apc Delta (KAD) Rats Are Susceptible to 4-NQO-Induced Tongue Carcinogenesis

**DOI:** 10.3390/cancers6031522

**Published:** 2014-07-21

**Authors:** Takuji Tanaka, Masahito Shimizu, Takahiro Kochi, Yohei Shirakami, Takayuki Mori, Naoki Watanabe, Takafumi Naiki, Hisataka Moriwaki, Kazuto Yoshimi, Tadao Serikawa, Takashi Kuramoto

**Affiliations:** 1 Department of Diagnostic Pathology (DDP) & Research Center of Diagnostic Pathology (RC-DiP), Gifu Municipal Hospital, 7-1 Kashima-Cho, Gifu 500-8513, Japan; E-Mail: naoki@watanabe.name; 2 Department of Tumor Pathology, Gifu University Graduate School of Medicine, 1-1 Yanagido, Gifu 501-1194, Japan; 3 Department of Internal Medicine/Gastroenterology, Gifu University Graduate School of Medicine, 1-1 Yanagido, Gifu 501-1194, Japan; E-Mails: shimim-gif@umin.ac.jp (M.S.); kottii924@yahoo.co.jp (T.K.); shirakamiyy@yahoo.co.jp (Y.S.); hmori@gifu-u.ac.jp (H.M.); 4 Department of Pharmacy, Ogaki Municipal Hospital, 4-86 Minaminokawa-cho, Ogaki 503-8502, Japan; E-Mail: ta_mori04@yahoo.co.jp; 5 Department of Clinical Laboratory, Gifu Municipal Hospital, 7-1 Kashima-cho, Gifu 500-8513, Japan; E-Mail: gnaiki-gif@umin.org; 6 The Institute of Laboratory Animals, Graduate School of Medicine, Kyoto University, Yoshidakonoe-cho, Sakyo-ku, Kyoto 606-8501, Japan; E-Mails: kyoshimi@anim.med.kyoto-u.ac.jp (K.Y.); serikawa@anim.med.kyoto-u.ac.jp (T.S.); tkuramot@anim.med.kyoto-u.ac.jp (T.K.)

**Keywords:** tongue, carcinogenesis, susceptibility, inflammation, *Apc* mutant rats

## Abstract

Despite widening interest in the possible association between infection/inflammation and cancer development, knowledge of this issue in relation to oral cancer remains inadequate. This study aimed to determine the susceptibility of *Apc*-mutant Kyoto Apc Delta (KAD) rats, which are vulnerable to developing inflammation-associated colorectal carcinogenesis, to 4-nitroquinoline 1-oxide (4-NQO)-induced tongue carcinogenesis in order to clarify the role of inflammation in oral cancer. KAD (20 males and 22 females) and F344/NS1c (22 males and 23 females) rats received drinking water with or without 4-NQO (20 ppm) for eight weeks. Histopathological and immunohistochemical analyses of the tongue were performed at week 20. Additionally, the mRNA expression of inflammatory cytokines in the tongue mucosa was determined at week 8. Tongue squamous cell carcinoma (SCC) developed in the KAD and F344/NS1c rats that received 4-NQO. Regardless of gender, the incidence and multiplicity of tongue SCC were greater in the KAD rats than in the F344/NS1c rats. In addition, the multiplicity of tongue SCC in the female KAD rats was significantly greater than that observed in the male KAD (*p* < 0.01) and female F344/NS1c rats (*p* < 0.05). The levels of inflammation and the mRNA expression of inflammatory cytokines in the tongue in the 4-NQO-treated female KAD rats were the highest among the rats given 4-NQO. These results show that KAD rats, particularly females, are susceptible to 4-NQO-induced tongue carcinogenesis, suggesting the utility of models employing KAD rats for investigating the pathobiology of oral (tongue) carcinogenesis associated with inflammation.

## 1. Introduction

Half of all causes of head and neck squamous cell carcinoma, which represents the fifth most frequent cancer worldwide, are located in the oral cavity. Approximately 1.6 million diagnoses and 333,000 deaths each year worldwide are attributable to head and neck squamous cell carcinoma [1]. Oral cancer is a multifactorial disease that is primarily associated with chronic tobacco and alcohol use. Chronic inflammation, viral (human papillomavirus) infection and genetic predispositions may also be involved in oral tumorigenesis [2]. Since the oral cavity is constantly exposed to environmental substances, including certain carcinogens, the tissue is frequently under inflammatory stress and particularly vulnerable to various infections causing infection/inflammation-related diseases, including cancer [2]. The specific mechanisms by which chronic inflammation causes oral carcinogenesis are yet not fully understood. Chronic inflammation can be induced by various factors, such as bacterial or viral infections or chemical irritants that persistently stimulate the immune system [3,4]. Malignant neoplasms are frequently surrounded by an inflammatory microenvironment rich in inflammatory cytokines, growth factors and chemokines, which promote malignant cell growth [5,6]. These factors are produced by the tumor itself and its surrounding tissue, and thus contribute to the progression of malignancy [7].

Since Dr. Rudolf Carl Virchow proposed that micro-inflammation resulting from irritation leads to most chronic diseases, including cancer [8,9], this concept has only recently been confirmed in experimental and clinical studies [6,7,10]. It is estimated that underlying infections and inflammatory reactions are linked to 15%–25% of all cancers. The role of chronic inflammation in carcinogenesis has been examined in studies of pro- and anti-inflammatory cytokines. Despite widening interest in the possible association between infection/inflammation and cancer development in several tissues [11,12,13], knowledge of this issue in relation to oral cancer remains inadequate. Indeed, oral inflammation may influence oral cancer development in the oral cavity [4,14,15,16]; however, no systemic studies have clarified this relationship using animal models.

Adenomatous polyposis coli (*APC*) is a 2,843-amino acid polypeptide composed of multiple domains. Most cancer-linked *APC* mutations occur in the central region of *APC* and result in truncation of almost half of the C terminal regions of the protein [17]. *APC* has been identified as the causative gene of familial adenomatous polyposis of the colon, which is characterized by the presence of numerous polyps in the large intestine [18]. Alteration of *APC* is also found in malignancies of other tissues [19,20], including the oral cavity [21,22,23,24,25]. Recently, we developed a mutant rat that carries a homozygous nonsense mutation at codon 2523 (*Apc^△2523^*), called the Kyoto Apc Delta (KAD) rat [26]. The KAD rat expresses truncated APC proteins that contain β-catenin binding sites but lack the C terminus (321 amino acids in length) able to bind to microtubules, EB1 and *Drosophila* disk large proteins. KAD rats survive to adulthood and are free of intestinal tumors. However, when given colitis-inducing agents, KAD rats exposed to a low dose of a colonic carcinogen, azoxymethane (AOM), exhibit a significantly higher incidence and multiplicity of colon tumors within a short-term period (at week 15) compared with that observed in control F344 rats [26,27]. On the other hand, KAD rats given AOM alone do not develop colon tumors by 15 weeks, as found in AOM-treated F344 rats [26]. These findings suggest that KAD rats are susceptible to inflammation-associated carcinogenesis in the colon [28].

In the current study, we aimed to determine whether KAD rats are susceptible to 4-nitroquinoline 1-oxide (4-NQO)-induced oral (tongue) carcinogenesis [29,30] in order to obtain a better understanding of the role of inflammation in the pathobiology of oral cancer. In addition, differences in the degree of tongue inflammation induced by 4-NQO between the KAD and F344 rats were estimated according to the mRNA expression levels of several inflammatory cytokines and inducible inflammatory enzymes and the results of immunohistochemistry using antibodies for several inflammatory biomarkers in the tongue.

## 2. Results

### 2.1. Preneoplastic and Neoplastic Lesions in the Tongue in the KAD and F344/NS1c Rats that Received 4-NQO

**Figure 1:**
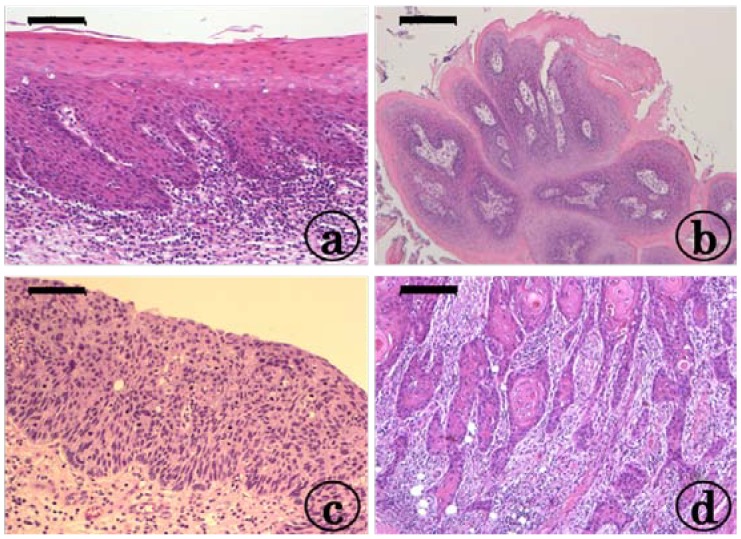
Representative photos of tongue lesions in the female KAD rats that received 20 ppm 4-NQO in drinking water for eight weeks. (**a**) Tongue dysplasia with moderate atypia; (**b**) squamous cell papilloma; (**c**) squamous cell carcinoma in situ; and (**d**) invasive squamous cell carcinoma (well differentiated). H&E staining, bars: (**a**) 100 μm, (**b**) 200 μm, (**c**) 100 μm and (**d**) 100 μm.

**Figure 2:**
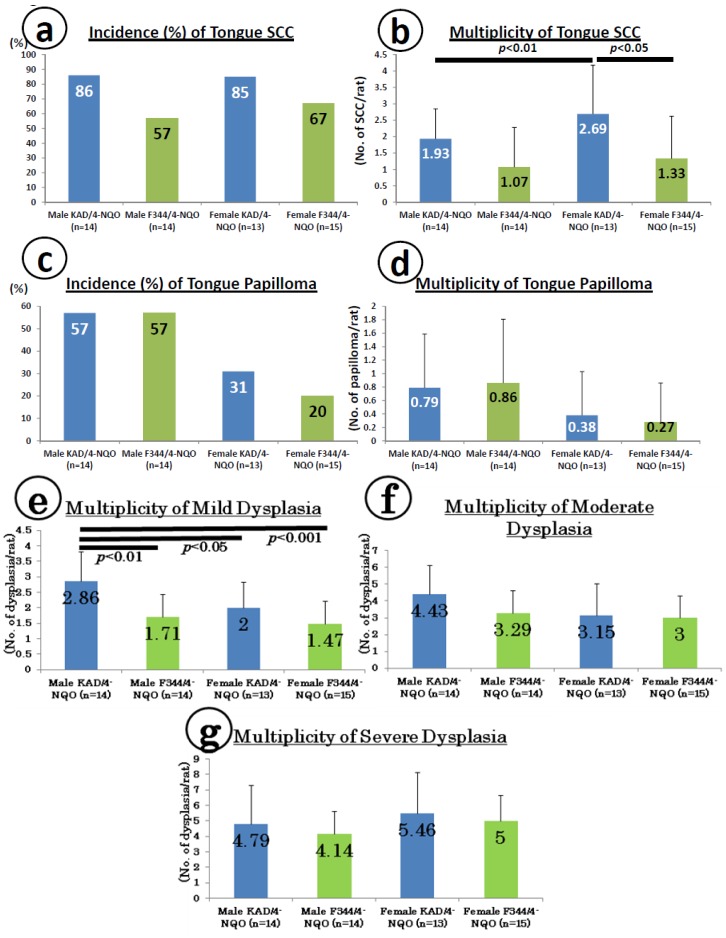
Incidence and multiplicity of tongue lesions in the KAD and F344/NS1c rats. (**a**) Incidence of tongue squamous cell carcinoma (SCC); (**b**) multiplicity of tongue SCC; (**c**) incidence of papilloma; (**d**) multiplicity of papilloma; (**e**) multiplicity of mild dysplasia; (**f**) multiplicity of moderate dysplasia; and (**g**) multiplicity of severe dysplasia.

At week 20, tongue preneoplastic lesions (Figure 1a), histopathologically confirmed squamous cell dysplasia and tumors, histopathologically confirmed squamous cell papilloma (PAP, Figure 1b) and SCC without invasion (Figure 1c) and with invasion (Figure 1d) developed in both the KAD and F344/NS1c rats that received 4-NQO for eight weeks. The incidence and multiplicity of tongue SCCs are shown in Figure 2a,b, respectively. The incidence of tongue SCC in the KAD rats of either sex (86% in males and 85% in females) was higher than that observed in the F344/NS1c rats (57% in males and 67% in females), although the differences were statistically insignificant. Similarly, the multiplicity of tongue SCC in the KAD rats of either sex (1.93 ± 0.92 in males and 2.69 ± 1.49 in females) was greater than that observed in the F344/NS1c rats (1.07 ± 1.21 in males and 1.33 ± 1.29 in females), while the values noted in the female KAD rats were significantly higher than those observed in the female F344/NS1c rats (*p* < 0.05). The multiplicity of tongue SCC of female KAD rats was significantly higher than that observed in the male KAD rats (*p* < 0.01).

The incidence (Figure 2c) and multiplicity (Figure 2d) of tongue PAP in both sexes of KAD (57% in males and 31% in females) and F344/NS1c (57% in males and 20% in females) rats were comparable, although the incidence of tongue PAP in the female KAD rats was slightly higher than that observed in the female F344/NS1c rats. For combined SCC and PAP (Figure 2e,f), the multiplicity of total tumors in the female KAD rats (3.08 ± 1.38, *p* < 0.05) was significantly higher than that observed in the female F344/NS1c rats (1.60 ± 1.55, Figure 2f). As to tongue dysplasia, such lesions developed in all KAD and F344/NS1c rats of both sexes (Figure 2g,h). Regarding the multiplicity of tongue dysplasia, the values in the KAD rats of both sexes were higher than those observed in the F344/NS1c rats without statistical significance (Figure 2h).

### 2.2. Inflammation in the Tongue in the KAD and F344/NS1c Rats that Received 4-NQO

**Figure 3:**
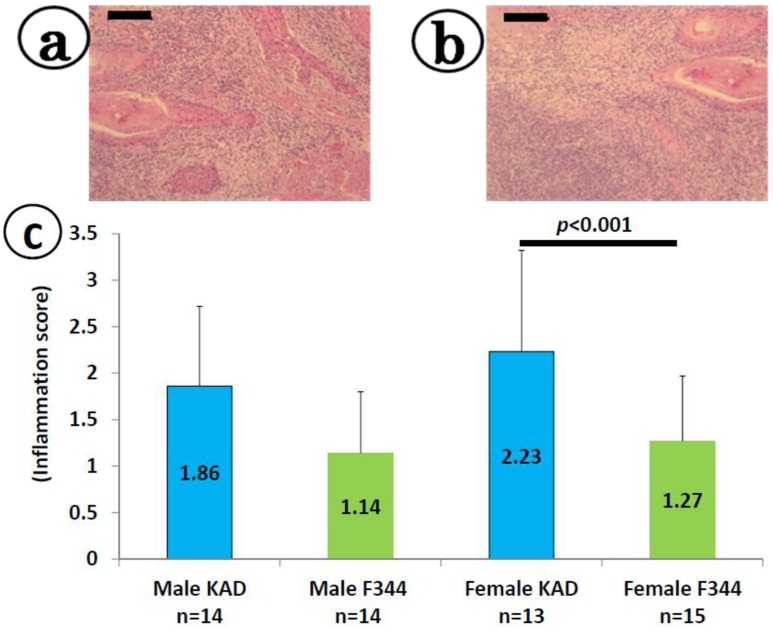
Inflammation scores for the tongue in the KAD and F344/NS1c rats. (**a**) and (**b**) are representative photos of tongue SCCs with inflammation in a male KAD and female KAD rat, respectively. (**c**) The values of the inflammation score represent the mean ± SD. (**a**) and (**b**) H&E staining, bars: 200 μm.

**Figure A1:**
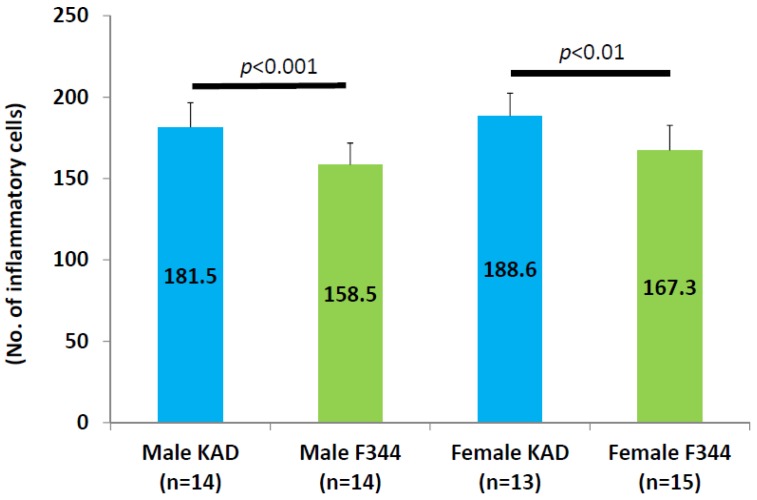
The number of inflammatory cells infiltrating the tongue (per microscopic field at ×400). The values are represented as the means ± SD.

We observed inflammatory cells (lymphocytes, neutrophils, macrophages, plasma cells, mast cells) in the tongue tissues with or without pathological lesions. As illustrated in Figure 3, the inflammation scores in the KAD rats (1.86 ± 0.86 in males and 2.23 ± 1.09 in females) were greater than those observed in the F344/NS1c rats (1.14 ± 0.66 in males and 1.27 ± 0.70 in females), while the values noted in the female KAD rats were significantly greater than those observed in the female F344/NS1c rats (*p* < 0.001). The results regarding the number of inflammatory cells infiltrating the tongue (per microscopic field at ×400) are summarized in Appendix Figure A1. The values in the male (181.5 ± 15.1) and female (188.6 ± 13.9) KAD rats were significantly higher than those observed in the male (158.5 ± 13) and female (167.3 ± 15.4) F344/NS1c rats, respectively (*p* < 0.001 for males and *p* < 0.01 for females).

### 2.3. Classification of Inflammatory Lymphocytes in the Tongue on Immunohistochemistry of CDs

**Figure A2:**
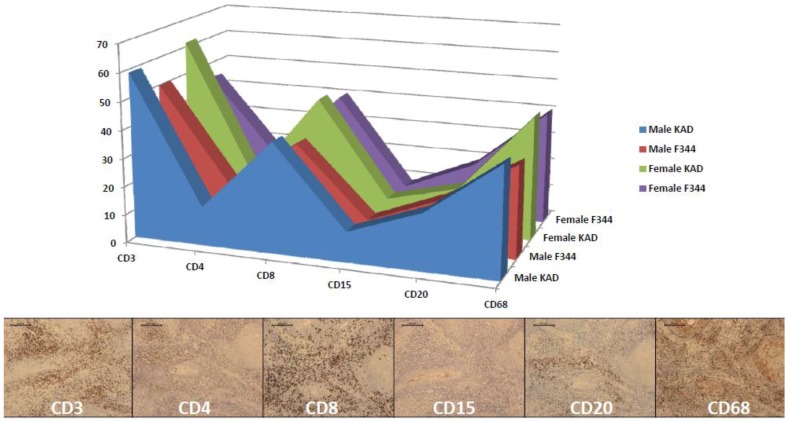
Number of inflammatory cells immunohistochemically positive for CD3, CD4, CD8, CD15, CD20 and CD68 in the tongue tissues of the KAD and F344/NS1c rats given 4-NQO. The values are represented as the means. The bars in the representative photos are 200 μm.

The degree of lymphocyte infiltration in the tongue in both the KAD and F344/NS1c rats was classified based on immunohistochemistry of CD3, CD4, CD8, CD15, CD20 and CD68. As shown in Appendix Figure A2, CD3 (T cell)-, CD8 (regulatory T cell)- and CD68 (macrophages and mast cells)-positive inflammatory cells were predominant among inflammatory cells. The distribution of these phenotypic inflammatory cells did not differ between the KAD and F344/NS1c rats or between males and females.

### 2.4. Mast Cell Density in the Tongue

**Figure 4:**
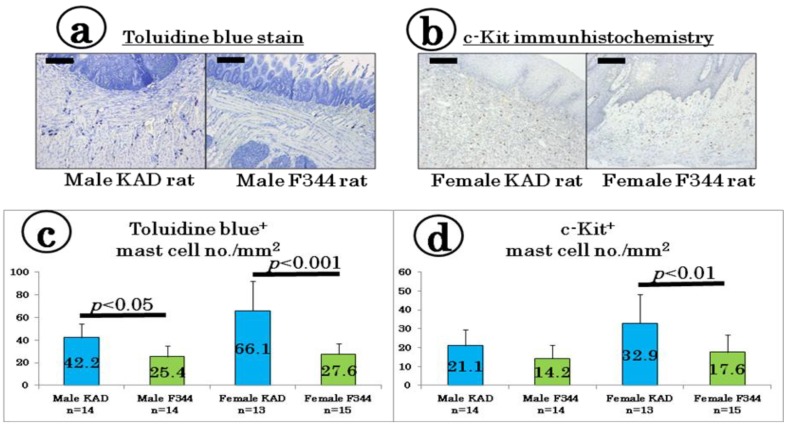
Presence of tongue mast cells determined using (**a**) toluidine blue staining and (**b**) c-Kit immunohistochemistry. Number of mast cells detected using (**c**) toluidine blue stain and (**d**) c-Kit immunohistochemistry. The values are presented as the mean ± SD. The bars in (**a**) and (**b**) are 200 μm.

We determined the mast cell density in the tongue tissues with or without tumors using toluidine blue staining (Figure 4a) and c-Kit-immunohistochemistry (Figure 4b). As shown in Figure 4c, the number of toluidine blue-positive mast cells in the male (42.2 ± 12.5) and female (66.1 ± 26.2) KAD rats was greater than that observed in the male (25.4 ± 9.6) and female (27.6 ± 9.5) F344/NS1c rats, respectively (*p <* 0.05 for males and *p* < 0.001 for females). In the c-Kit immunohistochemistry sections, the number of c-Kit-positive mast cells in the tongue tissues of the female KAD rats (32.9 ± 15.4) was significantly higher than that observed in the female F344/NS1c rats (17.6 ± 9.1, *p* < 0.01, Figure 4d).

### 2.5. Immunohistochemical Expression of Hif-1α and Nf-κB in the Tongue SCCs

**Figure 5:**
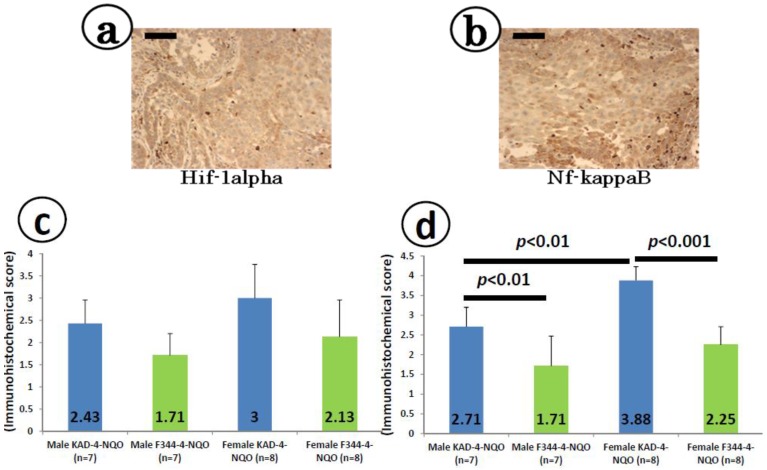
Immunohistochemistry for (**a**) Hif-1α and (**b**) Nf-κB in the tongue SCCs in the female KAD rats. Positive scores for (**c**) Hif-1α and (**d**) Nf-κB represent the mean ± SD. Inflammation scores for the tongue in the KAD and F344/NS1c rats. (**a**) and (**b**) are representative photos of tongue SCCs with inflammation in a male KAD and female KAD rat, respectively. (**c**) The values of the inflammation score represent the mean ± SD. (**a**) Hif-1α immunohistochemistry and (**b**) Nf-κB immunohistochemistry, bars: 100 μm.

The SCC cells were immunohistochemically positive for Hif-1α (Figure 5a) and Nf-κB (Figure 5b). As illustrated in Figure 5c, the scores for Hif-1α-positive SCC cells in the KAD rats of either sex were larger than those observed in the F344/NS1c rats, without statistical significance. On the other hand, the scores for NF-κB-positive SCC cells in the KAD rats of both sexes were significantly larger than those observed in the F344/NS1c rats (2.71 ± 0.49 *vs.* 1.71 ± 0.76, *p <* 0.01 for males and 3.88 ± 0.35 *vs.* 2.25 ± 0.96, *p* < 0.001 for females, Figure 5d). The values in the female KAD rats were significantly higher than those observed in the male KAD rats (*p* < 0.01, Figure 5d).

### 2.6. mRNA Expression Levels of Inducible Inflammatory Enzymes and Pro-Inflammatory Cytokines in the Tongue at Week 8

**Figure 6:**
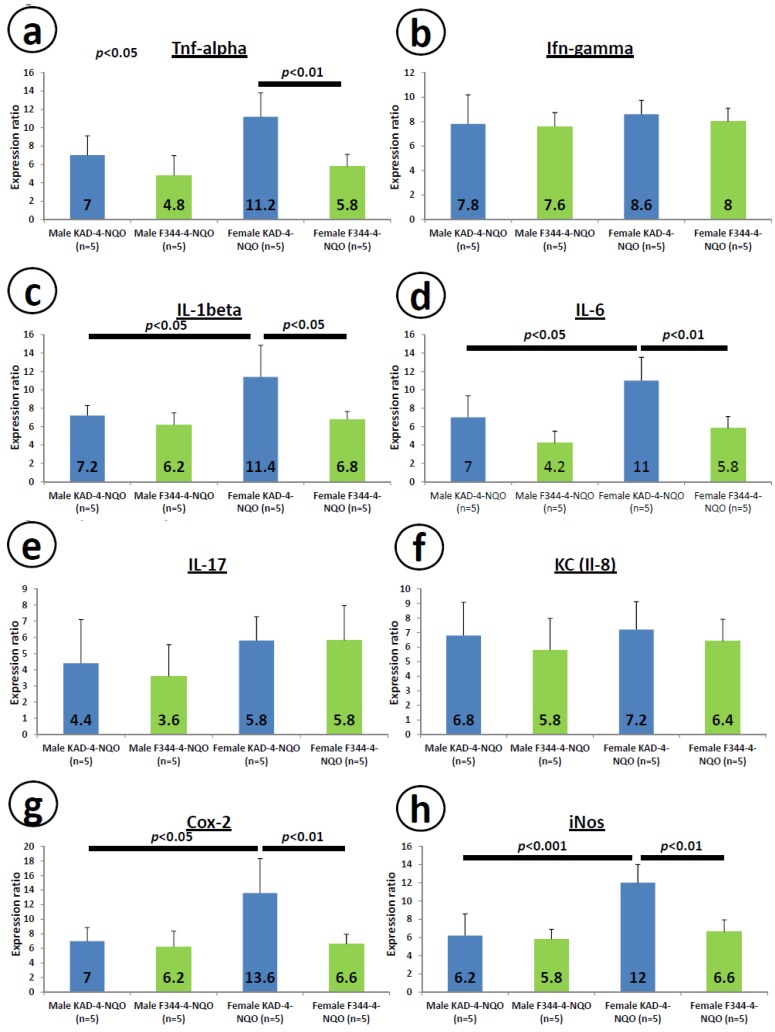
mRNA expression levels of pro-inflammatory cytokines (**a**–**f**) and inducible inflammation-related enzymes (**g**,**h**) in the tongue tissues of the KAD and F344/NS1c rats. Total RNA was extracted from the tongue tissues of untreated and 4-NQO-treated rats. The mRNA levels of (**a**) *Tnf-α*, (**b**) *Ifn-γ*, (**c**) *Il-1*β, (**d**) *Il-6*, (**e**) *Il-17*, (**f**) *KC* (*Il-8*), (**g**) *iNos* and (**h**) *Cox-2* were measured using quantitative RT-PCR. The expression levels were normalized to that of *β-actin* mRNA. The data are presented as the mean ± SD for three independent assays (n = 5 from each group). The ordinates show the relative mRNA expression (to *β-actin*).

Figure 6 illustrates the relative mRNA expression levels of *Tnf-α* (Figure 6a), *Ifn-γ* (Figure 6b), *Il-1β* (Figure 6c), *Il-6* (Figure 6d), *Il-17* (Figure 6e), *KC* (*Il-8*, Figure 6f), *Cox-2* (Figure 6g) and *iNos* (Figure 6h) in the tongue tissues at week 8. The expression levels of *Tnf-α* (Figure 6a), *Il-1β* (Figure 6c), *Il-6* (Figure 6d), *Cox-2* (Figure 6g) and *iNos* (Figure 6h) in the tongue were significantly greater in the female KAD rats than in the female F344/NS1c rats (*p* < 0.01 for *Tnf-α*, *Il-6*, *Cox-2* and *iNos*, and *p* < 0.05 for *Il-1β*). However, the expression levels of *Ifn-γ* (Figure 6b), *Il-17* (Figure 6e) and *KC* (*Il-8*, Figure 6f) did not differ significantly between the KAD and F344/NS1c rats.

## 3. Discussion

In the current study we showed that KAD rats, particularly females, are susceptible to 4-NQO-induced tongue carcinogenesis compared with controls, F344/NSlc rats. In addition, the inflammatory reactions were more severe in the KAD rats that received 4-NQO than the F344/NSlc rats that received 4-NQO. The immunohistochemical (c-Kit) and histochemical (toluidine blue stain) analyses revealed that the number of mast cells infiltrating the tongue was higher in the KAD rats than in the F344/NSlc rats, when the animals were given 4-NQO, although the distribution of T and B cell markers did not differ between the two strains. In addition, the mRNA expression levels of *Tnf-α*, *Il-1β*, *Il-6*, *Cox-2* and *iNos* in the tongue were greater in the KAD rats than in the F344/NSlc rats. In the tongue SCC lesions, the immunohistochemical expression of Nf-κB was prominent in the KAD rats compared to that observed in the F344/NSlc rats. These findings suggest that KAD rats are sensitive to inflammatory stimuli and susceptible to inflammation-related oral cancer development.

In the tongue tissues of the rats of both strains exposed to 4-NQO, numerous inflammatory cells had infiltrated the submucosa, where dysplastic and neoplastic lesions developed. The number of inflammatory cells in the KAD rats of either sex was higher than that observed in the F344/NSlc rats. In both strains, these cells predominantly included T cell types, as determined on immunohistochemistry. Oral lichen planus has been reported to be associated with an increased risk of oral SCC [31]. Considering that oral lichen planus is characterized by the presence of a T cell-mediated chronic immune-inflammatory reaction against an undefined antigen within the basal layer of the oral squamous epithelium and regulated by an upregulated expression of several inflammatory mediators [32], our present results are of interest. About 60% of the patients with lichen planus are women, our findings in the KAD rats given 4-NQO are interesting.

Other interesting findings of this study include the results showing that the immunohistochemical and histochemical analyses detected many mast cells in the submucosa of the tongue in the KAD rats of both sexes when compared to that observed in the F344/NSlc rats. Similar findings have been reported in the setting of human oral submucous fibrosis [33], a high-risk precancerous condition, and oral squamous cell carcinoma [34]. Although the role of mast cells in oral carcinogenesis is not fully understood, the actions of these cells are known to increase the risk of thus tumor angiogenesis [34,35], resulting in carcinogenesis progression [36], as documented in other tissues [37].

The immunohistochemical expression levels of *Hif-1α* and *Nf-κB* were estimated in the SCC tongue lesions that developed in the KAD and F344/NSlc rats in this study. Both parameters, with the levels of Nf-κB in particular, were greater in the KAD rats than in the F344/NSlc rats. Hif-1α is known to play an important role in the pathogenesis of oral cancer and functions an independent prognostic marker of oral SCC [38]. In addition, Nf-κB regulates many proteins involved in multiple stages of carcinogenesis in several tissues, including the oral cavity [39,40], and thus may also be a potential target molecule for chemoprevention of tongue cancer [41,42,43].

In the present study, we determined the mRNA expression levels of several cytokines (*Tnf-α*, *Ifn-γ*, *Il-1β*, *Il-6*, *Il-17* and *Il-8*) and inducible inflammatory enzymes (*Cox-2* and *iNos*) in the tongue tissues of the KAD and F344/NSlc rats that received 4-NQO and observed elevation in all parameters in the KAD rats, especially females, compared to that noted in the F344/NSlc rats. Elevation of the levels of *Tnf-α*, *Il-1β*, *Il-6*, *Cox-2* and *iNos* was also prominent, with statistical significance. These findings suggest that the presence of inflammation in the microenvironment has effects in promoting the formation and progression of tumors and KAD rats have also been reported to exhibit delayed repair against tissue injury due to prolonged inflammation and a reduced level of microvessel angiogenesis compared to that observed in F344/NSlc rats [28]. The serum levels of *Il-1β*, *Il-6* and *Tnf-α* are higher in patients with oral cancer than in healthy subjects [44]. Recent studies assessing the levels of several cytokines, including *Il-1β*, *Il-6*, *Il-8* and *Tnf-α* in saliva have identified a panel of pro-inflammatory cytokines as markers of oral malignancy [45,46]. In addition, several cytokines have been suggested to induce genomic instability [47]. Therefore, the increased expression of *Il-6* and *Tnf-α* observed in this study is interesting. As to the Cox-2 and iNos expression, our previous studies demonstrated that specific inhibitors of these inducible inflammatory enzymes are able to inhibit 4-NQO-induced tongue carcinogenesis [48,49,50], suggesting that these enzymes play a role in the development of tongue cancers and are potential targets for the prevention and treatment of oral carcinogenesis [29,30].

## 4. Experimental

### 4.1. Animals, Chemicals and Diet

F344/NS1c and KAD (homozygous for the *Apc^△2523^* mutation, official strain name: F344-*Apc^m1Kyo^*) rats of both sexes (5 weeks of age) were purchased from Japan SLC, Inc. (Hamamatsu, Japan). The KAD rats were backcrossed five times with female F344/NSIc rats to remove latent mutations induced by *N*-ethyl-*N*-nitrosourea [26]. The rats were kept in the animal facility of the Division of Animal Experiments, Life Science Research Center, Gifu University Graduate School of Medicine under conditions of controlled humidity (50% ± 10%), and temperature (23 ± 2 °C) with a 12/12-h light/dark cycle. The animals were housed in plastic cages with free access to a standard pellet diet (CRF-1, Oriental Yeast Co., Ltd, Tokyo, Japan) and tap water. After arrival, they were quarantined for the first seven days and randomized by body weight into experimental and control groups. 4-NQO (CAS no. 56-57-5, 98% pure), which was used to induce tongue tumor formation, was obtained from Wako Pure Chemical Inc. (Osaka, Japan), dissolved in tap water to a final concentration of 20 ppm and stored in a dark and cold room.

### 4.2. Experimental Protocol

A total of 60 KAD (30 males and 30 females) and 65 F344/NSlc rats (32 male and 33 females) were used in this study. Beginning at 6 weeks of age, 37 KAD rats (19 males and 18 females) and 39 F344/NS1c rats (19 males and 20 females) received 4-NQO (20 ppm) in drinking water for eight weeks followed by drinking water without 4-NQO for the subsequent 12 weeks. Twenty-three KAD (11 males and 12 females) and 26 F344/NSlc (13 males and 13 females) rats given tap water without 4-NQO served as the control group. All rats were carefully observed daily during the experiment. At week 8, five rats from each group were used to measure the mRNA expression levels of cytokines and inducible inflammatory enzymes in the tongue. The experiment was terminated 20 weeks after its initiation, and the remaining animals were sacrificed via deep ether anesthesia. At necropsy, the digestive organs, including the oral cavity, were carefully inspected for preneoplastic and neoplastic lesions and the number of grossly visible tumors in the tongue was recorded. The tongues were then processed for histopathological and immunohistochemical examinations after being fixed in 10% buffered formalin. On the histopathological analysis, the mast cell density and type of inflammatory cells were determined using histochemical and/or immunohistochemical stained sections of the tongue tissues. In addition, the mRNA expression levels of *Tnf-α*, *Il-1β*, *IL-6*, *Ifn-γ*, *IL-17*, *KC (Il-8)*, *Cox-2 and iNos* in the tongue mucosa of the KAD (n = 5 from each group) and F344 (n = 5 from each group) rats were determined at week 8.

### 4.3. Histopathological Diagnosis of the Tongue Lesions

After being fixed in 10% buffered formalin, the tongue tissues were embedded in paraffin wax blocks, and the histological sections (3 μm thick) were stained with hematoxylin and eosin (H&E) for histopathological diagnosis. The presence of epithelial lesions was diagnosed by a pathologist, T.T. [48]. Grading of dysplasia was performed based on the following criteria: mild dysplasia shows proliferation of dysplastic cells of the basal and parabasal layers which does not extend beyond the lower third of the tongue epithelium; moderate dysplasia shows proliferation of dysplastic cells extending into the middle one-third of the epithelium; and severe dysplasia shows abnormal proliferation from the basal layer into the upper third of the epithelium. Carcinoma in situ is composed with cancer cells in the whole epithelium but without invasion.

### 4.4. Inflammation Scoring of the Tongue with or without Preneoplastic and Neoplastic Lesions

The degree of inflammation of the tongue tissues with or without dysplasia and tumors was scored by a pathologist, T.T., based on the following grading criteria: Grade 0, scattered inflammatory cells; Grade 1, moderate inflammation and/or the presence of locally extensive loose infiltrates at the junction of the mucosa and submucosa; Grade 2, marked and multifocal inflammation and/or the presence of moderately dense infiltrates multifocally obscuring the junction of the mucosa and submucosa; Grade 3, marked and multifocal inflammation and/or the presence of moderately dense infiltrates multifocally obscuring the junction of the mucosa and submucosa; and Grade 4, marked and diffuse inflammation and/or the presence of dense infiltrates diffusely obscuring the junction of the mucosa and submucosa. Inflammation was scored in the whole tongue surface (mucosa and submucosa interface). Ten representative fields per side were examined at ×400 magnification under a light microscope.

### 4.5. Immunohistochemistry and Histochemistry

Paraffin-embedded sections of the tongues of the rats in all groups were used for the immunohistochemical and histochemical analyses. Serial histological sections (3 μm thick) were created from each paraffin wax block. Immunohistochemical staining was performed using an automated system (Ventana Benchmark XTsystem; Ventana, Touchstone, AZ, USA), according to the manufacturer’s instructions. Primary antibodies against CD3 (rabbit monoclonal, #790–4341; Roche Diagnostic K.K., Tokyo, Japan), CD4 (rabbit monoclonal, #790–4423; Roche Diagnostic K.K.), CD8 (rabbit monoclonal, #790–4460; Roche Diagnostic K.K.), CD15 (mouse monoclonal, #760–2504; Roche Diagnostic K.K.), CD20 (mouse monoclonal, #760–2531; Roche Diagnostic K.K.), CD68 (mouse monoclonal, #790–2931; Roche Diagnostic K.K.), c-Kit (rabbit monoclonal, #790–2222; Roche Diagnostic K.K.), hypoxia-inducible factor 1-α (Hif1-α, rabbit polyclonal, ab81634, 1:100 dilution; Abcam, Inc., Cambridge, MA, USA) and nuclear factor-kappaB (Nf-κB, rabbit monoclonal, ab32360, 1: 250 dilution; Abcam, Inc.) were used in this study. Solutions of CD3, CD4, CD8, CD15, CD20, CD68 and c-Kit for use in immunohistochemistry were purchased from the same company. In each case, the positive and negative controls were run concurrently. As the final step, the sections were lightly counterstained with Mayer’s hematoxylin (Merck, Tokyo, Japan).

The degree of immunoreactivity for the antibodies against Hif-1α and Nf-κB was assessed in the tongue squamous cell carcinoma (SCC) lesions using a microscope (Olympus BX41, Olympus Optical Co., Tokyo, Japan). The intensity and localization of immunoreactivity against the primary antibodies was scored with grading between 0 and 5 by a pathologist (T.T.) who was unaware of the treatment group to which the slide belonged: Grade 0, ~15% of the cancer cells showing positive reactivity; Grade 1, 16%–30% of the cancer cells showing positive reactivity; Grade 2, 31%–45% of the cancer cells showing positive reactivity; Grade 3, 46%–60% of the cancer cells showing positive reactivity; Grade 4, 61%–75% of the cancer cells showing positive reactivity; and Grade 5, ~75% of the cancer cells showing positive reactivity).

In order to detect mast cells, the sections were stained with 1% toluidine blue in addition to immunohistochemical staining with c-Kit. For the assessment of the mast cell density, the highest density of staining (hot spots) was determined on the initial scan obtained at low magnification (×100). The number of mast cells in five different fields was counted at ×400 magnification using a computer imaging analysis system (Leica QWin V3 image analysis software; Leica Microsystems, Heidelberg, Germany). For each slide, the number of mast cells in each field was counted, summed and then divided by the number of fields in order to obtain the mean value.

### 4.6. Total RNA Extraction and Quantitative Real-Time PCR

At week 8, five rats in each group were deeply anesthetized with ether and killed. Their tongues were used for quantitative real-time PCR of *Tnf-α*, *Il-1β*, *Il-6*, *Ifn-γ*, *Il-17*, *KC (Il-8)*, *Cox-2 and iNos*. The tissues were rapidly removed, dissected and stored at −80 °C. Total RNA was extracted from the tongue tissues using the RNeasy Mini Kit (Qiagen, Tokyo, Japan) according to the manufacturer’s protocol. cDNA was then synthesized from total RNA using the High-Capacity cDNA Reverse Transcription Kit (Applied Biosystems Japan Ltd., Tokyo, Japan). A quantitative real-time PCR analysis of individual cDNA was performed with an ABI Prism 7500 instrument (Applied Biosystems Japan Ltd.) using TaqMan Gene Expression Assays (Applied Biosystems Japan Ltd.). The primers sequences used in this study are listed in Appendix Table A1. The PCR cycling conditions were as follows: 50 °C for two minutes and 95 °C for 10 min, followed by 40 cycles of 95 °C for 15 s and 60 °C for one minute. The expression level of each gene was normalized to that of *β-actin* using the standard curve method. Each assay was performed in triplicate, and the average was calculated.

### 4.7. Statistical Analysis

The multiplicity (number of lesion/rat) of preneoplastic and neoplastic lesions and the scores obtained on the histological and immunohistochemical analyses were statistically analyzed using either the Tukey or Bonferroni multiple comparison posttest. The incidence of tongue lesions was compared between the groups using Fisher’s exact probability test. The statistical analysis of differences in the mRNA expression levels was performed using the Kruskal-Wallis test. Differences were considered to be statistically significant at a *p* value of < 0.05.

## 5. Conclusions

In conclusion, the present findings show that KAD rats, especially females, are susceptible to 4-NQO-induced tongue carcinogenesis, suggesting that KAD rats are useful for investigating the pathobiology of oral (tongue) carcinogenesis associated with inflammation.

## Appendix

**Table A1:** Primer sequences used for qRT-PCR.

Genes (species)	Forward primer (5' to 3')	Reverse primer (5' to 3')
Tnf-α (rat)	GGCAGGTCTACTTTGGAGTCATTGC	ACATTCGAGGCTCCAGTGAATTCGG
Il-1β (rat)	ACCTGCTAGTGTGTGATGTTCCCA	AGGTGGAGAGCTTTCAGCTCACAT
Il-6 (rat)	GAGGATACCACTCCCAACAGACC	AAGTGCATCATCGTTGTTCATACA
Il-17 (rat)	GTCAATGCGGAGGGAAAG	CACGAAGCAGTTTGGGAC
KC (Il-8) (human)	TTGGCAGCCTTCCTGATT	AACTTCTCCACAACCCTCTG
Inf-γ (rat)	AAAGACAACCAGGCCATCAGCAAC	TCTGTGGGTTGTTCACCTCGAACT
Cox-2 (rat)	GCATTCTTTGCCCAGCACTTCACT	TTTAAGTCCACTCCATGGCCCAGT
iNos (rat)	AGAGAGATCGGGTTCACA	CACAGAACTGAGGGTACA
β-actin (rat)	TGGAATCCTGTGGCATCCA	TAACAGTCCGCCTAGAAGCA
